# Light-activated quantum dot potentiation of antibiotics to treat drug-resistant bacterial biofilms†

**DOI:** 10.1039/d1na00056j

**Published:** 2021-04-21

**Authors:** Dana F. Stamo, Prashant Nagpal, Anushree Chatterjee

**Affiliations:** Chemical and Biological Engineering, University of Colorado Boulder Boulder CO 80303 USA chatterjee@colorado.edu; Antimicrobial Regeneration Consortium Boulder CO 80301 USA; Sachi Bioworks, Inc. Boulder CO 80301 USA; Quantum Biology, Inc. Boulder CO 80301 USA

## Abstract

CdTe-2.4 eV quantum dots (QDs) show excellent efficacy due to their tunability and photo-potentiation for sterilizing drug-resistant planktonic cultures without harming mammalian cells but this QD fabrication has not been tested against biofilms. While the QD attack mechanism—production of superoxide radicals—is known to stimulate biofilm formation, here we demonstrate that CdTe-2.4 eV QD-antibiotic combination therapy can nearly eradicate *Escherichia coli*, methicillin-resistant *Staphylococcus aureus*, and *Pseudomonas aeruginosa* biofilms. CdTe-2.4 eV QD versatility, safety, and ability to potentiate antibiotics makes them a potential treatment strategy for biofilm-associated infections.

## Abstract 2

CdTe-2.4 eV quantum dots (QDs) show excellent efficacy due to their tunability and photo-potentiation for sterilizing drug-resistant planktonic cultures without harming mammalian cells but this QD fabrication has not been tested against biofilms.

## Graphical abstract

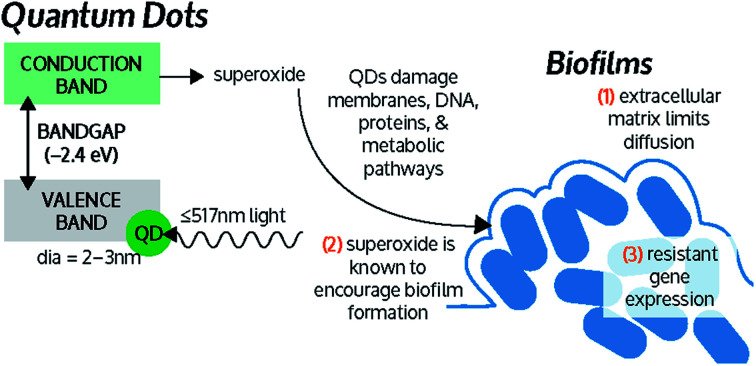

Antimicrobial resistance already threatens our ability to treat infections, perform surgery, and manage immunocompromising conditions, effects which are compounded by improper use of existing antibiotics and insufficient research into new treatments for multi-drug resistant (MDR) bacteria.^1–4^ Many treatment options are tested against planktonic bacterial cultures but at least 60% of clinical infections involve biofilms, a common bacterial growth form contributing to increased resistance to immune and antibiotic attack.^5–7^ Current strategies for biofilm-associated infections include antibiotic combinations or elevated doses, perpetuating the development of MDR bacteria while risking increased toxicity and secondary infections for the patient.^8,9^ These challenges underscore the need for alternative, dynamic therapies for MDR bacteria which are also capable of clearing bacterial biofilm-associated infections.^10^

Superoxide-generating light-activated quantum dots (QDs) can potentiate antibiotic treatments *in vitro* without harming mammalian cells.^11,12^ Upon QD absorption of a photon, a generated electron–hole pair collapses *via* an oxidation–reduction reaction, generating intracellular superoxide.^13^ Like macrophage oxidative burst, concomitant reactive oxygen species (ROS) damage cellular DNA and metabolic pathways.^14^ QD fabrication also allows for selection of materials, oxidation and reduction potentials, size, and surface chemistry, making QDs modifiable as needed to address a variety of infectious agents.^12^ Their small size and tunable properties facilitate diffusion through tissues and cellular uptake enabling unparalleled control over localized treatment.^12^ While various QD fabrications have been used as nanotherapeutics for eradication of bacterial biofilms, they are predominantly carbon-based and cannot be localized in the host to the site of infection.^15–19^ The cadmium telluride (CdTe) QDs characterized by Courtney *et al.* and used in these experiments are approximately 2–4 nm in diameter with a 2.4 eV bandgap and conduction band aligned with the reduction potential of dissolved oxygen.^11,12,20^ These features make CdTe-2.4 eV QDs excitable by ≤517 nm light to produce only localized superoxide which specifically targets bacteria.^11,13,21^ Only nanomolar concentration of CdTe-2.4 eV QDs are necessary to kill bacteria, making them safe and non-toxic to mammalian cells.^11,14,22,23^ The flexibility and safety of CdTe-2.4 eV QDs make them particularly well-suited for antimicrobial applications.

Despite the promise of CdTe-2.4 eV QDs, biofilms present unusual challenges. Resident bacteria diversify their gene expression—improving their response to environmental stressors such as antibiotic treatment through horizontal gene transfer of resistance genes—and surround themselves with an extracellular polymeric matrix, which may impede diffusion.^6,7,24^ The CdTe-2.4 eV QD killing mechanism (superoxide generation) also may encourage biofilm formation rather than eradication.^25–28^ Here, we explore the QD-biofilm interaction to reveal an alternative option for treating clinically-relevant bacterial infections that form biofilms.

We demonstrate synergy between 2.4 eV CdTe QDs and sub-Clinical & Laboratory Standards Institute (CLSI) breakpoint antibiotic treatments to early-stage, static *Escherichia coli* (*E. coli*) MG1655, methicillin-resistant *Staphylococcus aureus* (MRSA), and *Pseudomonas aeruginosa* (PAO1) biofilms. Used separately, effective concentrations of CdTe-2.4 eV QDs and antibiotics enhanced biofilm growth in clinical isolate strains compared to no treatment controls. Each strain, however, showed susceptibility to at least one CdTe-2.4 eV QD-antibiotic combination treatment (QD-ABX).

Biofilms were grown from 1 : 1 × 10^5^ overnight cultures for 48 hours in 96-well U-bottom plates as described by O'Toole^29^ in conditions ideal for each strain (ESI Table S1†), summarized in Fig. 1a. The biofilms were incubated with treatment brought to concentration in growth medium at 37 °C then illuminated with white LED-light for 8 hours to activate the CdTe-2.4 eV QDs. Post-treatment wells were screened for viability *via* (1) crystal violet (CV) staining, (2) 2 hour incubation with Resazurin metabolic assay, or (3) counting colony forming units (CFU) (Fig. 1b). Normalized Relative Fluorescence Units (RFU), CFU, and CV absorbances were calculated relative to untreated controls for each strain, respectively.

**Fig. 1:**
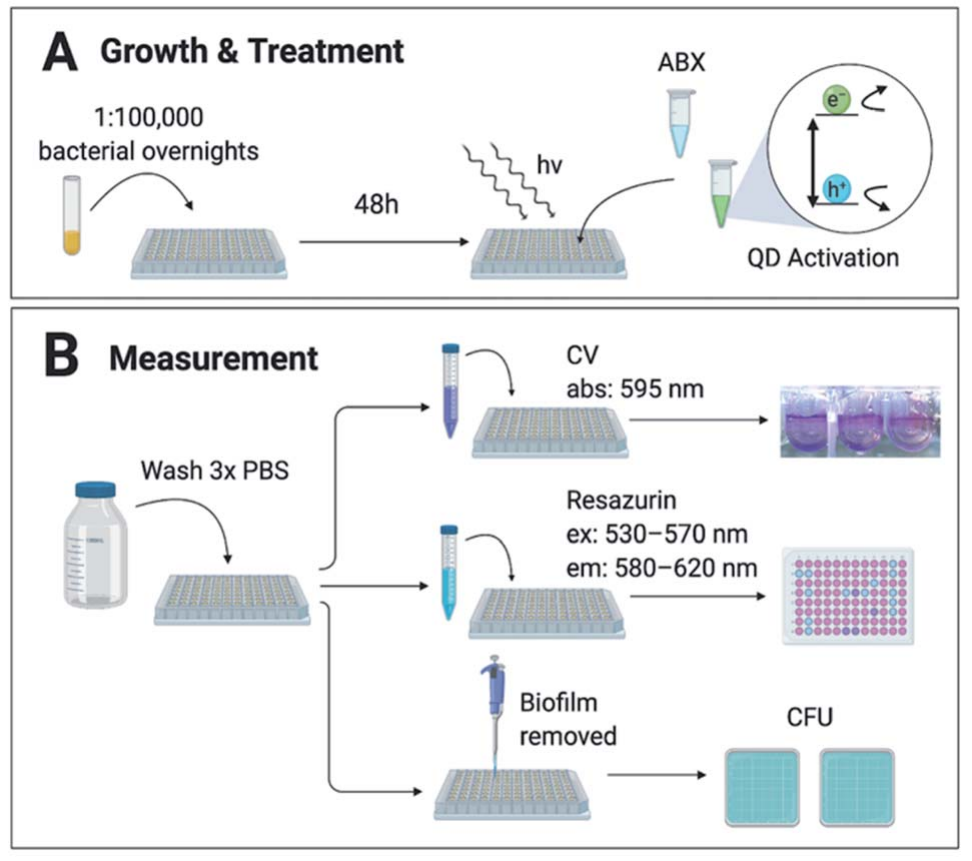
Method & experimental design. (A) Biofilms were grown for 48 hours from 1 : 1 × 10^5^ dilutions of each bacterial strain. Antibiotics (ABX) and CdTe-2.4 eV quantum dot (QD) treatments in growth medium were added for 8 hours with white LED light to activate the QDs. (B) Post-treatment wells were rinsed 3 times with phosphate-buffered saline (PBS) to remove planktonic cells and waste. Biofilms were measured *via* 1 of 3 methods: (1) crystal violet (CV) staining, (2) Resazurin metabolic assay, or (3) counting colony forming units (CFU). CV stains were solubilized in 70% ethanol and absorbance (abs) measured at 595 nm. Biofilms metabolized Resazurin for 2 hours before being measured at excitation (ex) 530–570 nm and emission (em) 580–620 nm. Absorbance and fluorescence was measured with a TECAN GENios Microplate reader. Biofilms were manually scraped off of each well using a pipette tip, then diluted in PBS and plated for CFU.

We used *E. coli* MG1655—a well-established model strain—to inspect correlation among the 3 biofilm viability assays. A significant correlation (*R* = 0.88993, *p* = 0.01751) between normalized RFU and CFU measurements for increasing CdTe-2.4 eV QD doses, provides evidence for the use of Resazurin as an accurate, high-throughput assay of biofilm viability (Fig. 2a). While CV-stained post-treatment biofilms show a trend consistent with that captured by Resazurin, CV stains any organic matter, resulting in artificially elevated measurements that did not represent viable cells. We analyzed QD-ABX synergy with *S*-values, which were calculated using the Bliss independence model by subtracting normalized RFU of QD-ABX measurements from the product of their component monotherapies (*S* > 0 indicates synergy, *S* < 0 indicates antagonism).^30–34^

**Fig. 2:**
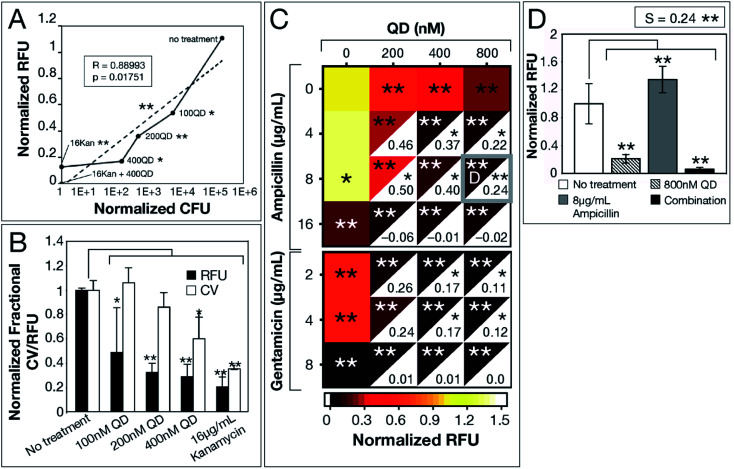
CdTe-2.4 eV QD treatment of *E. coli* MG1655 biofilm. Normalized 2 hour RFU and CFU were determined from the ratio of fluorescence of treatment conditions relative to the no treatment control. (A) Normalized CFU and RFU data of 48 hour biofilms post-treatment show correlation, indicating Resazurin is a viable measurement of biofilm viability. (B) CV staining shows a similar trend to normalized RFU, however, CV is not as sensitive due to its staining of extracellular material. (C) Heatmap shows biofilm viability post-treatment, where darker maroon indicates lower cell viability. Synergy *S*-values (calculated by subtracting RFU of the combination therapy from the product of RFU of its component monotherapies) are indicated within the white, bottom, right corners of each combination respectively. Full bar plots corresponding to each combination are shown in ESI Fig. S1, S2 and Table S2.† (D) Comparison of normalized RFU of the combination therapy and component monotherapies corresponding to the most statistically significant *S*-value (highlighted in **(**C**)** with a gray box). Treatment *p*-values were calculated with respect to the no treatment controls and synergy *p*-values were calculated with respect to the product of component monotherapies. These *p*-values are indicated by asterisks (1 asterisk = *p* ≤ 0.02, 2 asterisks = *p* ≤ 0.001). Data shown is an average of five biological replicates and error bars represent standard deviation. [Abbreviations: Relative Fluorescence Units (RFU), Colony Forming Units (CFU), Crystal Violet (CV), CdTe-2.4 eV quantum dots (QD), Kanamycin (Kan)].

CdTe-2.4 eV QD-monotherapies were effective for *E. coli* MG1655 showing dose-dependent effects (Fig. 2a–c). An 800 nM CdTe-2.4 eV QD dose cleared biofilms nearly to the same degree as 16 μg mL^−1^ kanamycin. Though low doses of CdTe-2.4 eV QD (100–200 nM) and antibiotic (4–8 μg mL^−1^ ampicillin, 2–4 μg mL^−1^ gentamicin) monotherapies achieved little killing (Fig. 2c), in combination their effects were amplified, significantly killing 48 hour biofilms with high synergy. Overall, *E. coli* MG1655 *S*-values were small since biofilms responded well to monotherapies (Fig. 2c). Fig. 2d shows that QD-ABX can negate the biofilm stimulation of sub-breakpoint ampicillin monotherapy. Though *E. coli* MG1655 already responded to CdTe-2.4 eV QD and antibiotic monotherapies, we achieved more robust killing with lower QD-ABX combination. We confirmed that the QD-ABX treatments were killing resident bacteria rather than triggering dispersal by testing the viability of media post-treatment (Fig. S2–S5†).

After demonstrating experimental proof-of-concept with *E. coli* MG1655, we tested QD-ABX on clinical isolates MRSA (for its relevance in dermal infections) and PAO1 (for its prevalence in lung infections).^35,36^ Bacteria establish biofilms in response to environmental stressors, including ROS. MRSA established significantly larger biofilms with CdTe-2.4 eV QD or sub-breakpoint gentamicin treatments compared to the untreated control (Fig. 3a). In combination, however, CdTe-2.4 eV QDs with 2 μg mL^−1^ gentamicin significantly reduced MRSA biofilm mass with high synergy (*S* = 2.46 in Fig. 3b).

**Fig. 3:**
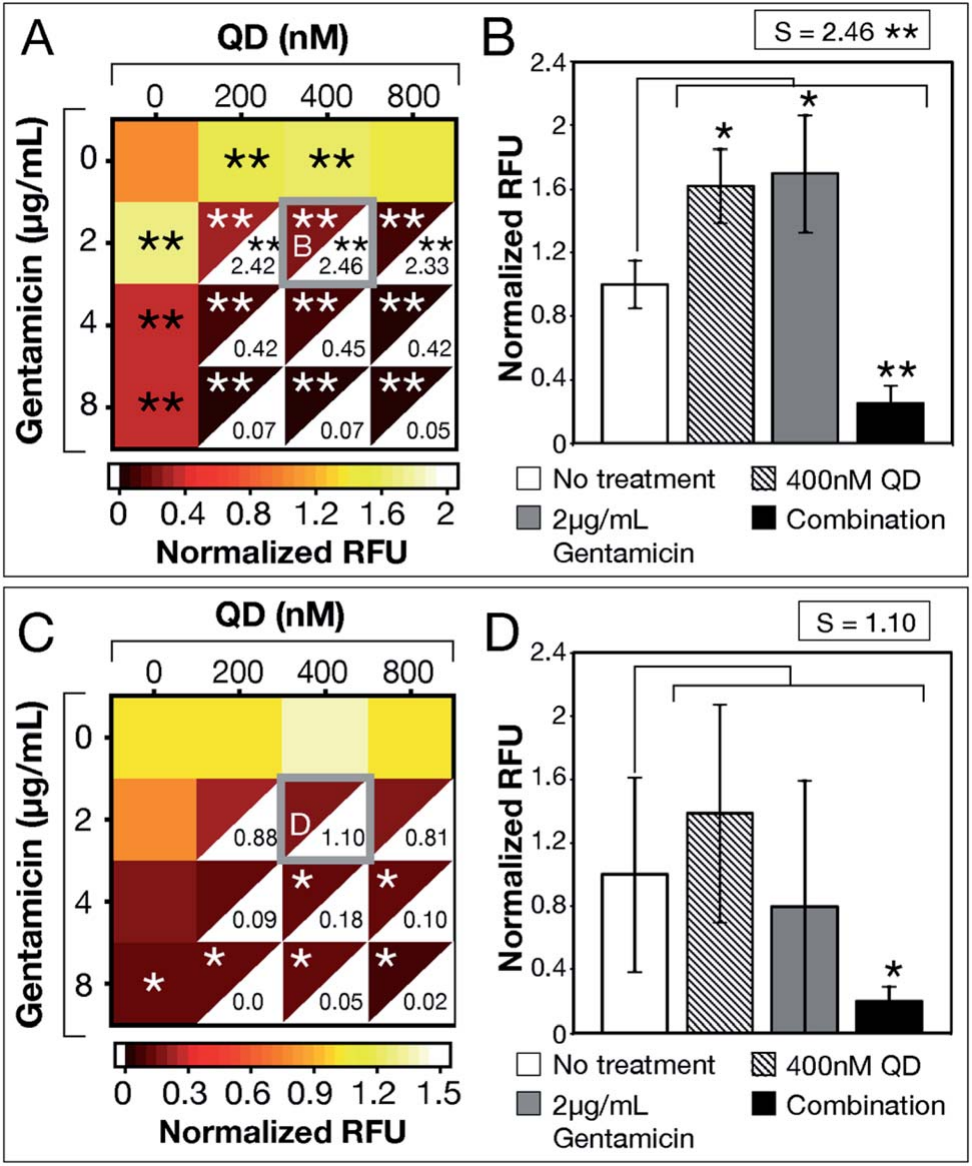
CdTe-2.4 eV QD treatment of MRSA and PAO1 biofilms**.** Normalized RFU (ratio of fluorescence of treatment conditions relative to no treatment for each strain, respectively) shows post-treatment biofilm viability for (A) MRSA and (C) PAO1, where darker maroon indicates lower viability. Synergy *S*-values (calculated by subtracting RFU of the combination therapy from the product of RFU of its component monotherapies) are indicated within the white, bottom, right corners of each combination respectively. Full bar plots corresponding to each combination are shown in ESI Fig. S6 and Table S3† for MRSA and ESI Fig. S10 and Table S4† for PAO1. Comparisons of normalized RFU of the combination therapies and component monotherapies corresponding to the most statistically significant *S*-values (highlighted in (A) and (C) with a gray box) are shown for (B) MRSA and **(**D) PAO1. Note that component monotherapies induce significant biofilm growth for MRSA biofilms rather than eradication. The large error bar associated with 2 μg mL^−1^ gentamicin monotherapy in panel (D) shows the unpredictable efficacy of low antibiotic dosages against PAO1 biofilms, which is resolved by combination therapy. Treatment *p*-values were calculated with respect to the no treatment controls and synergy *p*-values were calculated with respect to the product of component monotherapies. These *p*-values are indicated by asterisks (1 asterisk = *p* ≤ 0.02, 2 asterisks = *p* ≤ 0.001). Data shown is an average of five biological replicates and error bars represent standard deviation. [Abbreviations: Relative Fluorescence Units (RFU), CdTe-2.4 eV quantum dots (QD)].

CdTe-2.4 eV QD-monotherapy showed no significant variation in PAO1 biofilms (Fig. 3c). Furthermore, sub-breakpoint doses of gentamicin (2 μg mL^−1^) showed dramatic variation (visualized in Fig. 3d by the large error associated with 2 μg mL^−1^ gentamicin monotherapy), suggesting that effects of antibiotic monotherapy may vary with the bacterial population, making their efficacy difficult to predict. QD-ABX showed far more consistent results, even at mild concentrations, with reasonably high *S*-values.

As tested antibiotic concentrations increased, *S*-values decreased as antibiotic monotherapies were sufficient for biofilm clearance. For both clinical isolates, CdTe-2.4 eV QDs potentiated lower concentrations of antibiotics to eradicate established biofilms. Similar to *E. coli* MG1655, we confirmed killing rather than dispersal of biofilm bacteria by measuring the viability of post-treatment media (MRSA in Fig. S7–S9, PAO1 in Fig. S11–S13†). CdTe-2.4 eV QD and sub-breakpoint monotherapies risk inadvertent biofilm stimulation, but CdTe-2.4 eV QD-antibiotic potentiation eliminates this concern, with synergy suggesting enhancement or reversal of CdTe-2.4 eV QD or sub-breakpoint antibiotic monotherapies.

CdTe-2.4 eV QDs show potential not only for alternative therapies but also sterilization of surfaces prone to biofilm growth (such as faucets and implanted medical devices). This research lays a foundation for future work in treating late-stage biofilms with flow (to more accurately model clinical and industrial conditions). CdTe-2.4 eV QDs are activated by visible light, which limits their application to surface infections. Future work will explore the application of near-infrared light-activated indium phosphide quantum dots to establish similar foundational work for the treatment of deep-tissue biofilm-associated infections.^37^ QD-ABX eradicate biofilms with milder dosages (protecting patient microbiomes) and holistic disruption of cellular function (slowing MDR development). The versatility, safety, and ability to potentiate antibiotics makes CdTe-2.4 eV QDs a prime therapeutic candidate for persistent bacterial biofilm-associated infections.

## Author contributions

D. F. S. conducted all experiments. A. C. provided samples of MRSA and MG1655. D. F. S. and A. C. analyzed the results and wrote the manuscript. All authors edited the manuscript and have given final approval to the final version of the manuscript.

## Conflicts of interest

A. C. and P. N. have a patent on QD technology. D. F. S. declares no competing interests.

## Supplementary Material

NA-003-D1NA00056J-s001
