# Supplementary material for: Light-activated quantum dot potentiation of antibiotics to treat drug-resistant bacterial biofilms
Source: Nanoscale Adv. 2021 Apr 21;3(10):2782–6. doi: 10.1039/d1na00056j (PMC9419411; doi:10.1039/d1na00056j)
Supplement: NA-003-D1NA00056J-s001 [file NA-003-D1NA00056J-s001.pdf]

Electronic Supplementary Material (ESI) for Nanoscale Advances.
This journal is © The Royal Society of Chemistry 2021

Supplemental Information for:

Light-Activated Quantum Dot Potentiation of Antibiotics to Treat Drug-Resistant Bacterial Biofilms

Dana F. Stamo[1], Prashant Nagpal[1,2,4,5,6], and Anushree Chatterjee[1,3,4,5*]

[1]Chemical and Biological Engineering, University of Colorado Boulder, Boulder, CO

[2]Renewable and Sustainable Energy Institute, University of Colorado Boulder, Boulder, CO

[3]Biomedical Engineering, University of Colorado Boulder, Boulder, CO

[4]Antimicrobial Regeneration Consortium, Boulder, CO

[5]Sachi Bioworks, Inc., Boulder, CO

[6]Quantum Biology, Inc., Boulder, CO

Supplementary Materials and Methods:

Bacterial Cell Culture:

The *Escherichia coli* (*E. coli*) lab strain K12 (MG1655) and clinical isolate strain of *Pseudomonas aeruginosa* PAO1 provided by Dr. Michael Schurr of the University of Colorado Anschutz were maintained as a freezer stocks in 50% lysogeny broth (LB) (Sigma-Aldrich, St. Louis, MO) medium and 50% glycerol (50% solution with double de-ionized water) at –80˚C. Experimental replicates were streaked on solid LB containing 1.5% agar (VWR Chemicals, Ballycoolin, Dublin, Ireland) and incubated statically at 37˚C for 14–18 hours; PAO1 was plated on media also containing 1 mg/mL Ampicillin sodium salt (Sigma-Aldrich, St. Louis, MO). Each biological replicate was grown *in vitro* by adding one single colony forming unit from the solid plate medium to 1 mL of liquid LB. The liquid cultures were grown for 14–18 hours at 37˚C with shaking immediately prior to biofilm growth.

The clinical isolate strain methicillin-resistant *Staphylococcus aureus* (MRSA) (gifted from Dr. Nancy Madinger at University of Colorado Anschutz) was freezer-preserved in 50% Cation-adjusted Mueller Hinton Broth (CAMHB) (Becton, Dickinson and Company, Sparks, MD) medium and 50% glycerol (50% solution with double de-ionized water) at –80˚C. Experimental replicates were streaked on solid CAMHB with 1.5% agar and 1 mg/mL Ampicillin then incubated statically at 37˚C for 14–18 hours. Each biological replicate was grown as for *E. coli* MG1655 and PAO1 but replaced LB with CAMHB liquid media.

PAO1 and MRSA are Bio-Safety Level 2 strains and must be handled in a biosafety cabinet with personal protective equipment.

Biofilm Growth:

The overnight cultures for each biological replicate were diluted $1{:}1\times10^5$ in liquid LB for *E. coli* MG1655 and PAO1 and liquid CAMHB for MRSA. 100 µL of the diluted overnight cultures were added to a well in a 96-well U-bottom plate (Greiner Bio-One, Monroe, NC) for each experimental condition. The wells on the outermost perimeter of each plate were filled with water to prevent liquid media from evaporating during incubation. The plates were covered and incubated for 48 hours statically at 37˚C.

E. coli MG1655 and MRSA biofilms were given fresh media after 24 hours of growth. The old media was aspirated out, then 120 µL of liquid LB (for *E. coli* MG1655) or CAMHB (for MRSA) was added to each well. The additional volume was necessary to fully cover the air-liquid interface (where the biofilm forms) and ensure nutrients reach the 24-hour biofilm. PAO1 did not need this fresh media and grew for 48 hours, uninterrupted.

Quantum Dot Synthesis:

Quantum dot synthesis took place in a chemical safety hood while wearing personal protective equipment as some reagents pose risk of damage to organic tissue. Nitrogen gas was bubbled through 40 mL of double de-ionized water (dd-H_2O) to degas it; the tube was capped with an autoclaved septum with a needle poked through it to vent. 33 mg (0.93 mmol) of sodium borohydride ($NaBH_4$) (Fisher Chemical, Fair Lawn, NJ) were dissolved in 1 mL of degassed dd-H_2O in a 1.5 mL vial. Taking care not to expose the reaction to ambient oxygen, the $NaBH_4$-water solution was then mixed with 39 mg (0.31 mmol) tellurium (Te) powder (–325 mesh, Alfa Aesar, Ward Hill, MA) in a 2 mL vial with a septum cap (Thermo Scientific, Rockwood, TN) and a needle inserted through the septum to vent. The solution was reacted for 90 minutes, after which bubbling ceased and the solution turned an optically clear magenta, to produce the Te precursor solution.

To make a cadmium (Cd) precursor solution, 16 mg (2.2 mmol) of cadmium chloride ($CdCl_2$) (Aldrich Chemistry, St. Louis, MO) and 7.2 µL of 3-mercaptopropionic acid (MPA) (Sigma-Aldrich, St. Louis, MO) are dissolved into another 40 mL of degassed, dd-H_2O. 900 µL of 0.5 M sodium hydroxide (NaOH) (Fisher Chemical, Fair Lawn, NJ), 22.5 mL of degassed dd-H_2O, 22.5 mL of Cd precursor solution, and 75 µL of the Te precursor solution are mixed to give the final quantum dot (QD) solution.

A 500 µL aliquot of the final QD solution was added to a 1.5 mL PCR tube (USA Scientific, Ocala, FL) then held at 98˚C (in 30 second increments) in a thermocycler (Mastercycler Personal, Eppendorf, Hauppauge, NY) for 1–20 minutes to determine the batch-specific reaction time. Tubes were optically inspected under ultraviolet light to determine approximate reaction end point, then size was confirmed by measuring absorbance at 515 nm using a UV-vis spectrophotometer (UV-1600PC, VWR, Radnor, PA). The QD solution was wrapped in aluminum foil, sealed with tape, and stored at –20˚C.

Within 12 hours prior to treatment, QDs were reacted in a thermocycler as described above for the determined batch reaction time, then washed and filtered using a 10 kDa filter (Pierce™, Thermo Scientific, Rockford, IL). Prior to QD filtration, the filter was sterilized by centrifuging (Allegra X-14R Centrifuge, Beckman Coulter) it for 10 minutes at 4,000 RPM with 1 mL of 70% ethanol. The filter was then coated to stabilize QDs by centrifuging 2 mL of sterile pH 11 water for 10 minutes at 4,000 RPM. The entire volume of QDs was then centrifuged for 30 minutes at 4,000 RPM to remove any unreacted QDs, this was followed by two rinses of the QDs, first 5 mL of pH 11 water for 30 minutes at 4,000 RPM, then 2 mL of pH 11 water for 15 minutes at 4,000 RPM. An aliquot of the final retentate was used to determine QD concentration

by measuring absorbance at 515 nm with a UV-vis spectrophotometer. QD treatments were brought to concentration in CAMHB for MRSA and LB for *E. coli* MG1655 and PAO1.

Treatment:

Once biofilms had grown for 48-hours, they were carefully aspirated then rinsed (by pipetting up and down at least 6 times) 3 times with phosphate-buffered saline (PBS) (Thermo Fisher Scientific, Waltham, MA). Quantum dot and antibiotics Gentamicin sulfate (Lonza, Biowhittaker Reagents, Walkersville, MD), Ampicillin sodium salt, and Kanamycin sulfate (Sigma-Aldrich, St. Louis, MO) were brought to concentration in CAMHB for MRSA and LB for *E. coli* MG1655 and PAO1. 150 µL of treatment were added to each well to ensure full coverage of the air-liquid interface where the biofilm formed. Treated plates were incubated statically at 37˚C for at least 8 hours with ≤517 nm wavelength light to activate QDs. Post-treatment wells were prepared for viability assays by first transferring the post-treatment media to separate plates (for planktonic viability measurements) then rinsing (by pipetting up and down at least 6 times) 3 times with PBS to ensure all planktonic cells and wastes were washed out of the biofilm.

Crystal Violet Assay:

175 µL of 0.1% Crystal violet (CV) solution (Sigma-Aldrich, St. Louis, MO) was added to each freshly rinsed well and left to sit for 10 minutes at room temperature. The CV was aspirated then each well rinsed with PBS 3 times to remove any lingering CV stain. If photographs were taken of the stained biofilms, they were captured using the built-in camera feature of an iPhone X.

200 µL of 70% ethanol were added to each well and left for 10 minutes at room temperature to solubilize the CV stain. After 10 minutes, the contents of each well were

transferred to a corresponding 96-well, clear, flat-bottom plate (Greiner Bio-One, Monroe, NC), excepting the wells on the outermost perimeter. The absorbance of each well was measured using a platereader (TECAN™ GENios® Microplate Reader) at 550 nm.

Resazurin Metabolic Assay:

Resazurin stock solution (100x) was made by dissolving 0.5 g of Resazurin sodium salt (Sigma-Aldrich, St. Louis, MO) in 100 mL of PBS. This stock solution was diluted 1:100 with LB media for *E. coli* MG1655 and PAO1 and CAMHB for MRSA. For biofilms, 175µL of the diluted Resazurin was added to each freshly washed well and, for planktonic media, 1µL of diluted Resazurin was added to 99µL aliquot from each well, then incubated for 3 hours statically at 37˚C. The fluorescence was measured using a platereader (TECAN™ GENios® Microplate Reader) with excitation at 530–570 nm and emission at 580–620 nm.

Counting Colony Forming Units:

Freshly rinsed, post-treatment biofilms were scraped off manually using a pipette tip filled with 200 µL of PBS. Once scraped, the PBS and biofilm cells were pipetted up and down vigorously at least 10 times to fully incorporate cells from the pipette tip with the PBS in the well. The cells from each well are serially diluted, plated and incubated statically at 37˚C for 16 hours, then colony forming units are counted and used to determine cell load in each biofilm. 200 µL of 0.1% CV was added to each scraped well for 10 minutes, then aspirated and rinsed 3 times with PBS to visually ensure scraping was thorough.

Statistical Analysis and Data Visualization:

Data was analyzed using Microsoft Excel, p-values were determined using two-tail t-test for pairwise comparisons and two-factor ANOVA for combination treatment analysis. Heatmaps in figures 2c, 3a, and 3c were generated using Origin (OriginLab). Figure 1 was created using

BioRender. Supplemental Tables S1–4 were made in Microsoft Excel. Supplemental figures S3–6, S8–10, and S12–14 were made in GraphPad Prism 8. All figures were compiled in Adobe Illustrator 2021.

Strain	Streaking Conditions		Biofilm Conditions		
	Media	Antibiotics	Media	Temperature	Fresh Media at 24h
MG1655	LB	none	LB	37˚C	Yes
MRSA	CAMHB	1 mg/mL Amp	CAMHB	37˚C	Yes
PA01	LB	1mg/mL Amp	LB	25˚C	No

Supplemental Table S1: Ideal Growth Conditions. Each strain was streaked and allowed to form a biofilm under unique conditions in order to maximize biofilm growth. Strains were plated (for single colony replicates and post-treatment CFU) on either Lysogeny broth (LB) or cation-adjusted Mueller-Hinton broth (CAMHB) media agar. All streaked plates were incubated at 37˚C. Clinical isolates were plated on 1mg/mL Ampicillin (Amp) to control growth. Each strain was set to grow a biofilm in the liquid form of its same respective growth medium at either 37˚C or 25˚C (room temperature). Wells were aspirated and replaced with fresh media after biofilms had been growing for 24 hours for MG1655 and MRSA.

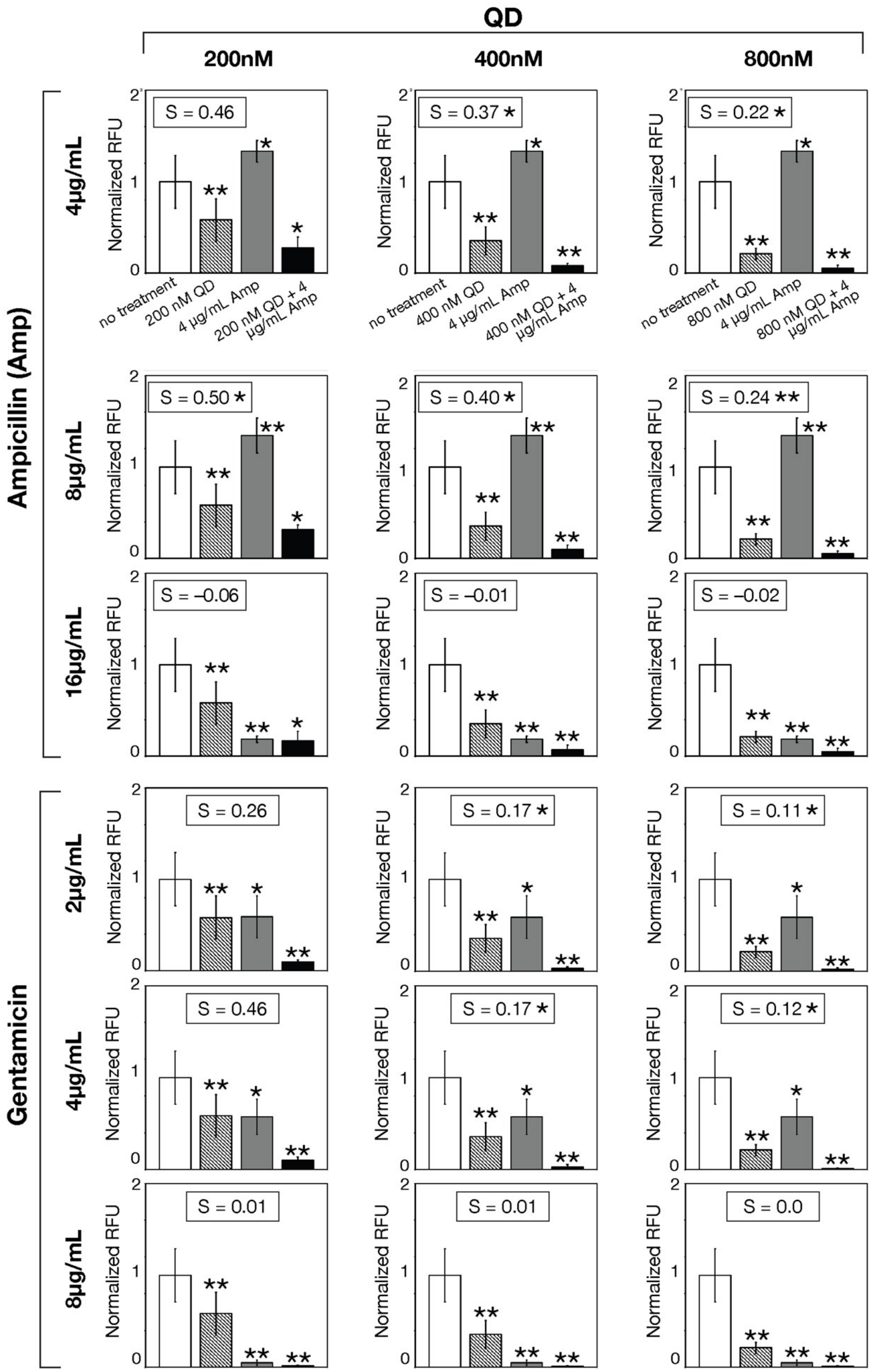
QD
200nM
400nM
800nM
Ampicillin (Amp)
4µg/mL
S = 0.46
S = 0.37 *
S = 0.22 *
Normalized RFU
no treatment
200 nM QD
4 µg/mL Amp
200 nM QD + 4 µg/mL Amp
400 nM QD
400 nM QD + 4 µg/mL Amp
800 nM QD
800 nM QD + 4 µg/mL Amp
8µg/mL
S = 0.50 *
S = 0.40 *
S = 0.24 **
16µg/mL
S = –0.06
S = –0.01
S = –0.02
Gentamicin
2µg/mL
S = 0.26
S = 0.17 *
S = 0.11 *
4µg/mL
S = 0.46
S = 0.17 *
S = 0.12 *
8µg/mL
S = 0.01
S = 0.01
S = 0.0

Supplemental Figure S1: MG1655 Combination-Monotherapy Comparison. This figure shows the full bar graphs of the relative fluorescence units (y-axis) of each combination therapy tested for MG1655 showing no treatment control (white), quantum dot (QD) (hatched), antibiotic (gray), and combination (black). Normalized resazurin fluorescence with respect to the no treatment control (1 asterisk: p-value ≤ 0.05, 2 asterisks: p-value ≤ 0.01). Synergy S-values (calculated by subtracting normalized relative fluorescence units of combination measurements from the product of its component monotherapies ($S>0$ indicates synergy, $S<0$ indicates antagonism)) are listed for each combination respectively. Antagonism occurred when monotherapy antibiotic or QD treatment decreased biofilm fluorescence alone but, in combination, significantly increased biofilm fluorescence compared to the no treatment control, suggesting that certain kinds of stress enhance biofilm formation. Synergy p-values listed alongside the S-value were calculated with respect to the product of component monotherapies. Data is an average of five biological replicates and error bars represent the standard deviation.

MG1655					
			QD		
			200nM	400nM	800nM
Ampicillin	4μg/mL	*S*	0.46	0.37	0.22
		p	0.131	0.045	0.228
	8μg/mL	*S*	0.5	0.4	0.24
		p	0.049	0.029	0.323
	16μg/mL	*S*	–0.06	–0.01	–0.02
		p	0.022	0.007	0.106
Gentamicin	2μg/mL	*S*	0.26	0.17	0.11
		p	0.056	0.066	0.081
	4μg/mL	*S*	0.46	0.17	0.12
		p	0.039	0.034	0.193
	8μg/mL	*S*	0.01	0.01	0
		p	0.043	0.02	0.192

Supplemental Table S2: *E. coli* MG1655 Combination Treatment S-values and Corresponding p-values. Synergy S-values (calculated by subtracting normalized relative fluorescence units of combination measurements from the product of its component monotherapies (S>0 indicates synergy, S<0 indicates antagonism)) are listed for each combination respectively. Synergy p-values listed alongside the S-value were calculated with respect to the product of component monotherapies using a paired t-test. Data is an average of four biological replicates and error bars represent the standard deviation.

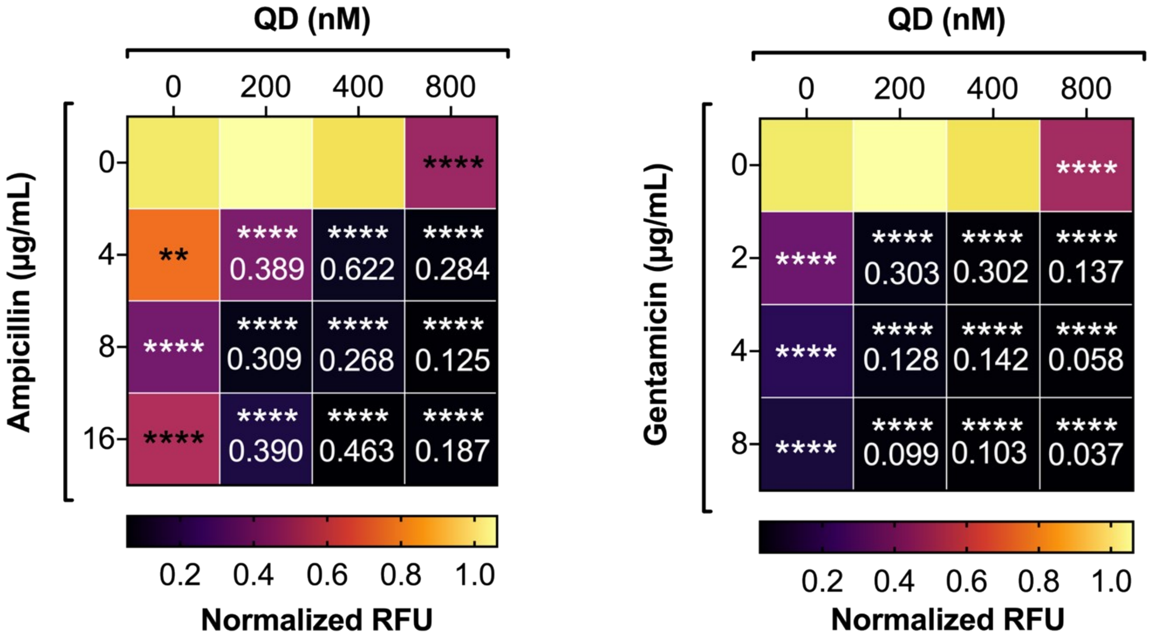

Supplemental Figure S2: *E. coli* MG1655 Planktonic Viability Post-treatment. Normalized RFU (ratio of fluorescence of treatment conditions relative to no treatment control) shows viability of planktonic cells in culture medium post-treatment corresponding to cells in Figure 2C. Synergy S-values calculated using a Bliss Independence model (subtracting RFU of combination therapy from the product of RFU of its component monotherapies) are listed for each combination in their respective cells. Treatment p-values were calculated using a two-factor ANOVA test relative to the no treatment control in the top left corner for each antibiotic combination respectively. P-values are indicated with asterisks (1 asterisk = $p \leq 0.0332$, 2 asterisks = $p \leq 0.0021$, 3 asterisks = $p \leq 0.0002$, 4 asterisks = $p \leq 0.0001$). Data shown is an average of 4 biological replicates and error bars represent standard deviation. [Abbreviations: Relative Fluorescence Units (RFU), CdTe-2.4 eV quantum dots (QD)]

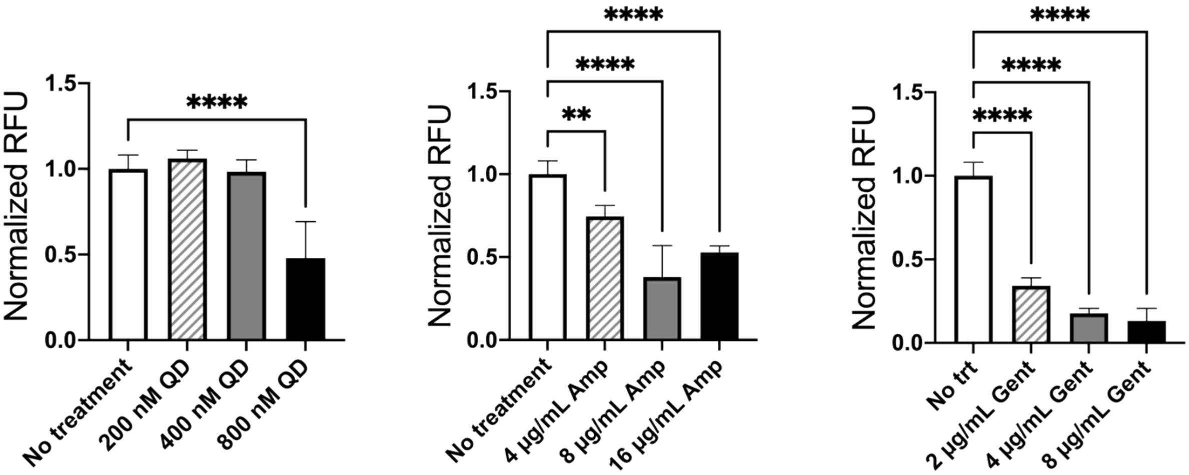

Supplemental Figure S3: ***E. coli*** **MG1655 Planktonic Viability Post-Monotherapeutic Treatment.** Normalized RFU (ratio of fluorescence of treatment conditions relative to no treatment control) shows viability of planktonic cells in culture medium post-treatment corresponding to cells in Figure 2C. Treatment p-values were calculated using a two-tailed t test relative to the no treatment control. P-values are indicated with asterisks (1 asterisk = $p \leq 0.0332$, 2 asterisks = $p \leq 0.0021$, 3 asterisks = $p \leq 0.0002$, 4 asterisks = $p \leq 0.0001$). Data shown is an average of 4 biological replicates and error bars represent standard deviation. [Abbreviations: Relative Fluorescence Units (RFU), CdTe-2.4 eV quantum dots (QD), Ampicillin (Amp), Gentamicin (Gent)]

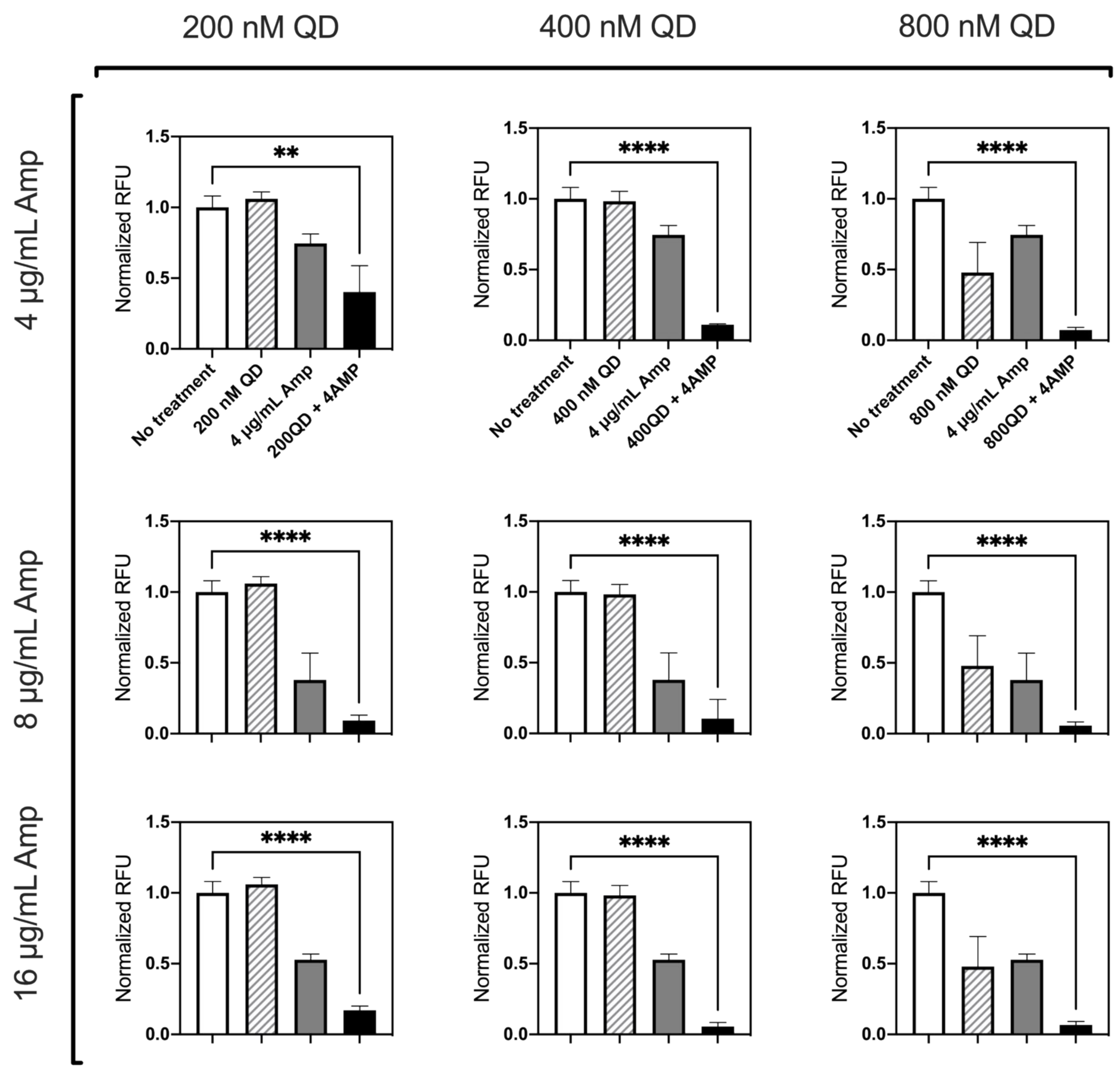

Supplemental Figure S4: *E. coli* MG1655 Planktonic Viability Post-Combination Therapy. Normalized RFU (ratio of fluorescence of treatment conditions relative to no treatment control) shows viability of planktonic cells in culture medium post-treatment corresponding to biofilms in Figure 2C and S2. Treatment p-values were calculated using a two-factor ANOVA test relative to the no treatment control. P-values are indicated with asterisks (1 asterisk = $p \leq 0.0332$, 2 asterisks = $p \leq 0.0021$, 3 asterisks = $p \leq 0.0002$, 4 asterisks = $p \leq 0.0001$). Data shown is an

average of 4 biological replicates and error bars represent standard deviation. [Abbreviations: Relative Fluorescence Units (RFU), CdTe-2.4 eV quantum dots (QD), Ampicillin (Amp)]

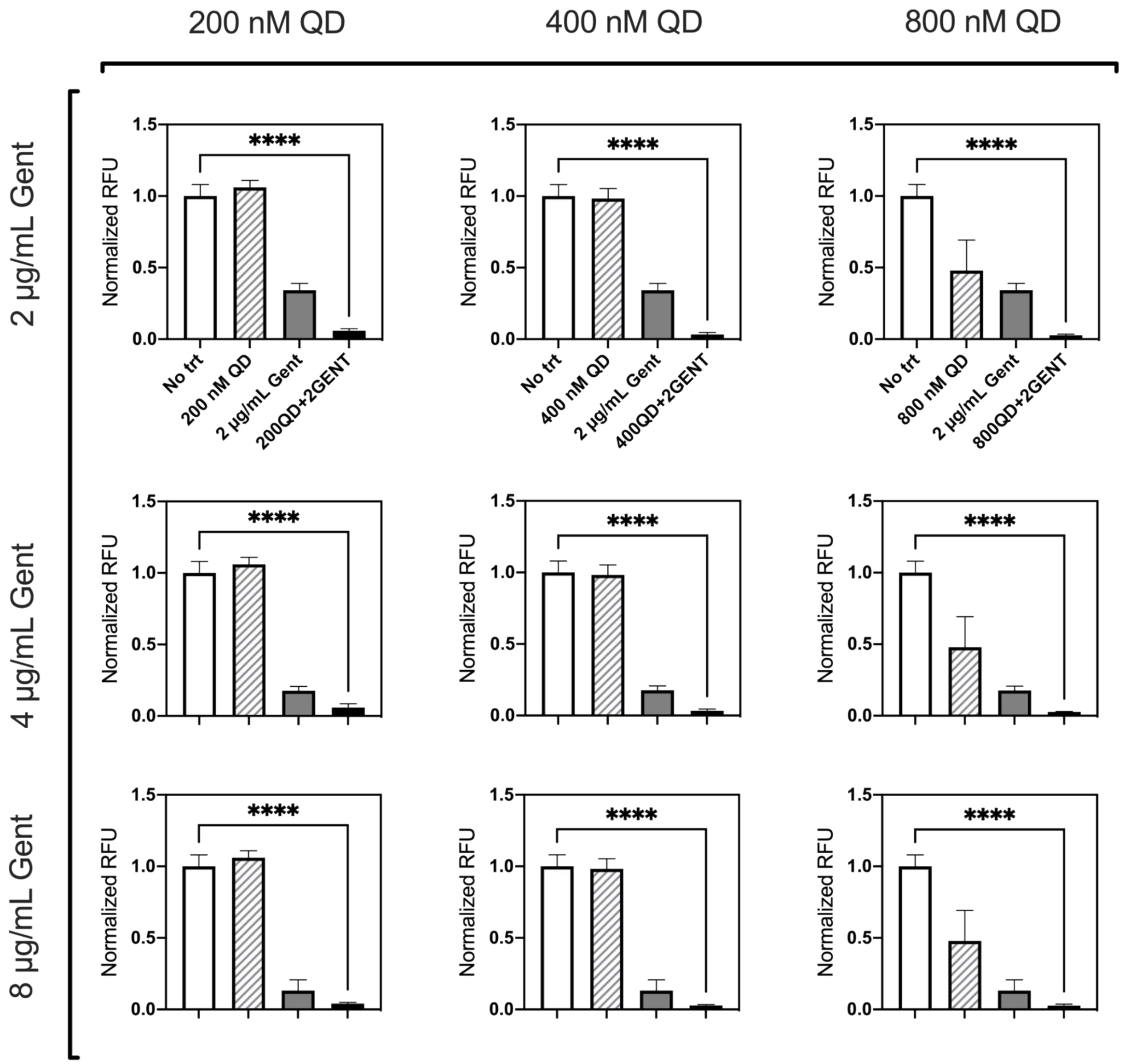

Supplemental Figure S5: ***E. coli*** **MG1655 Planktonic Viability Post-Combination Therapy.** Normalized RFU (ratio of fluorescence of treatment conditions relative to no treatment control) shows viability of planktonic cells in culture medium post-treatment corresponding to biofilms in Figure 2C and S2. Treatment p-values were calculated using a two-factor ANOVA test relative to the no treatment control. P-values are indicated with asterisks (1 asterisk = $p \leq 0.0332$, 2 asterisks = $p \leq 0.0021$, 3 asterisks = $p \leq 0.0002$, 4 asterisks = $p \leq 0.0001$). Data shown is an

average of 4 biological replicates and error bars represent standard deviation. [Abbreviations: Relative Fluorescence Units (RFU), CdTe-2.4 eV quantum dots (QD), Gentamicin (Gent)]

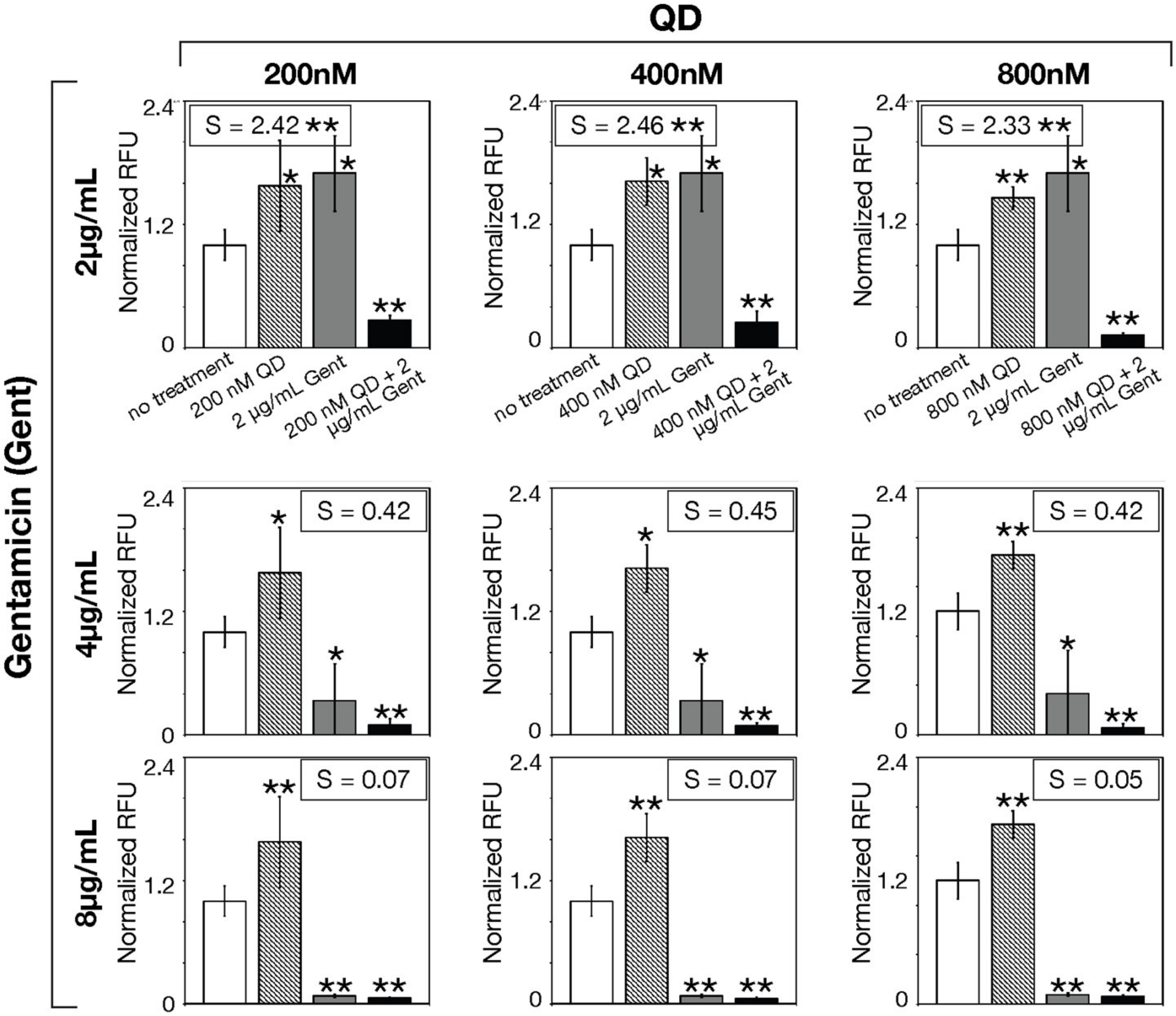

Supplemental Figure S6: MRSA Combination-Monotherapy Comparison. This figure shows the full bar graphs of the relative fluorescence units (y-axis) of each combination therapy tested for MRSA showing no treatment control (white), quantum dot (QD) (hatched), antibiotic (gray), and combination (black). Normalized resazurin fluorescence with respect to the no treatment control (1 asterisk: p-value ≤ 0.05, 2 asterisks: p-value ≤ 0.01). Synergy S-values (calculated by subtracting normalized relative fluorescence units of combination measurements from the product of its component monotherapies (S>0 indicates synergy, S<0 indicates

antagonism)) are listed for each combination respectively. Synergy p-values listed alongside the S-value were calculated with respect to the product of component monotherapies. Data is an average of five biological replicates and error bars represent the standard deviation.

MRSA					
			QD		
			200nM	400nM	800nM
Gentamicin	2μg/mL	*S*	2.42	2.46	2.33
		p	0.009	0.088	0.046
	4μg/mL	*S*	0.42	0.45	0.42
		p	0.001	0.089	0.001
	8μg/mL	*S*	0.07	0.07	0.05
		p	0.001	0.085	0.007

Supplemental Table S3: MRSA Combination Treatment S-values and Corresponding p-values. Synergy S-values (calculated by subtracting normalized relative fluorescence units of combination measurements from the product of its component monotherapies (S>0 indicates synergy, S<0 indicates antagonism)) are listed for each combination respectively. Synergy p-values listed alongside the S-value were calculated with respect to the product of component monotherapies. Data is an average of five biological replicates and error bars represent the standard deviation.

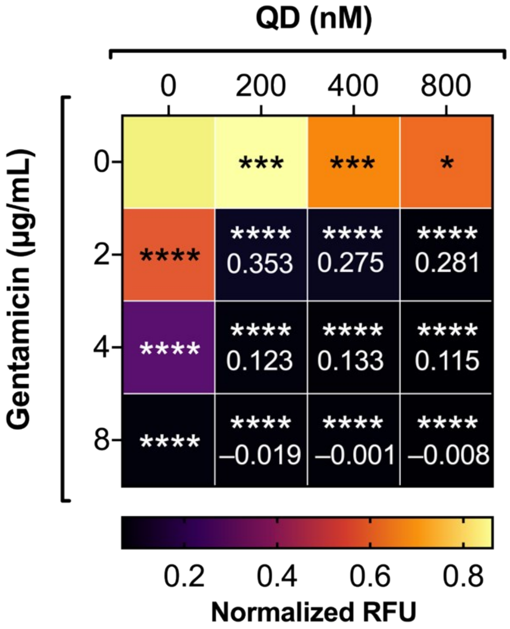

Supplemental Figure S7: MRSA Planktonic Viability Combination Therapy Heatmap. Normalized RFU (ratio of fluorescence of treatment conditions relative to no treatment control) shows viability of planktonic cells in culture medium post-treatment corresponding to cells in Figure 3A. Synergy S-values calculated using a Bliss Independence model (subtracting RFU of combination therapy from the product of RFU of its component monotherapies) are listed for each combination in their respective cells. Treatment p-values were calculated using a two-factor ANOVA test relative to the no treatment control in the top left corner. P-values are indicated with asterisks (1 asterisk = $p \leq 0.0332$, 2 asterisks = $p \leq 0.0021$, 3 asterisks = $p \leq 0.0002$, 4 asterisks = $p \leq 0.0001$). Data shown is an average of 4 biological replicates and error bars represent standard deviation. [Abbreviations: Relative Fluorescence Units (RFU), CdTe-2.4 eV quantum dots (QD)]

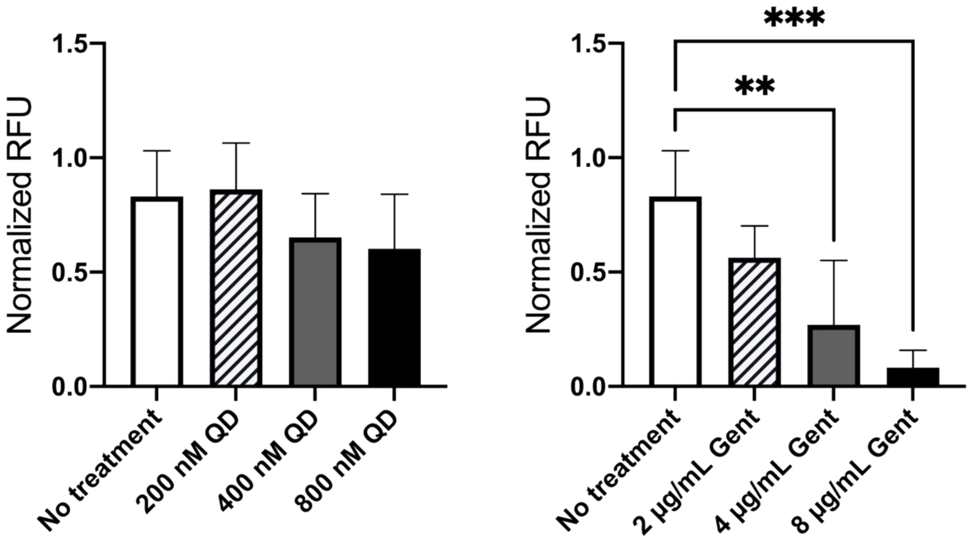

Supplemental Figure S8: MRSA Monotherapeutic Planktonic Viability. Normalized RFU (ratio of fluorescence of treatment conditions relative to no treatment control) shows viability of planktonic cells in culture medium post-treatment corresponding to cells in Figure 3A. Treatment p-values were calculated using a two-tailed t test relative to the no treatment control. P-values are indicated with asterisks (1 asterisk = $p \leq 0.0332$, 2 asterisks = $p \leq 0.0021$, 3 asterisks = $p \leq 0.0002$, 4 asterisks = $p \leq 0.0001$). Data shown is an average of 4 biological replicates and error bars represent standard deviation. [Abbreviations: Relative Fluorescence Units (RFU), CdTe-2.4 eV quantum dots (QD), Gentamicin (Gent)]

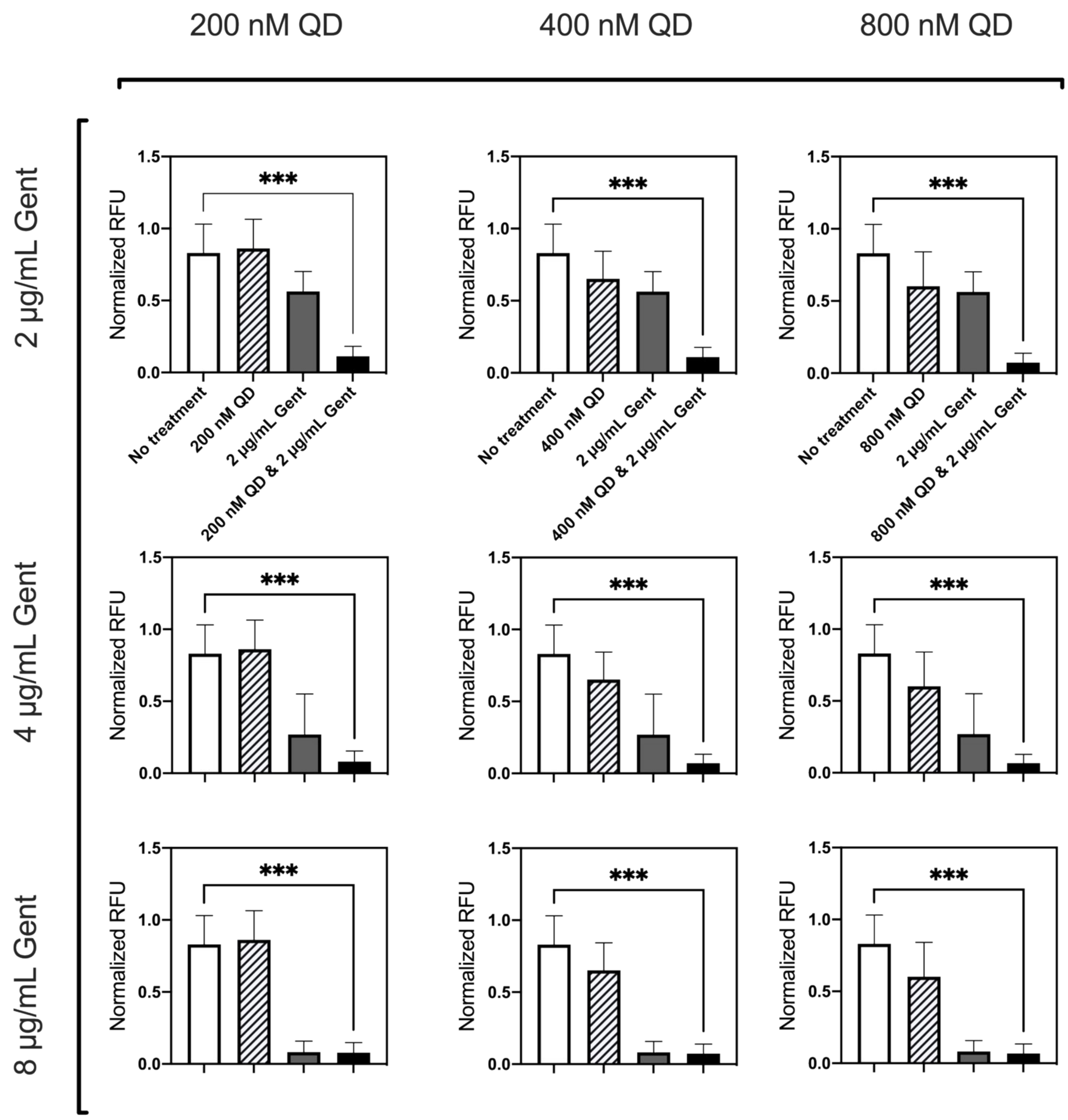

Supplemental Figure S9: MRSA Planktonic Viability Post-Combination Therapy. Normalized RFU (ratio of fluorescence of treatment conditions relative to no treatment control) shows viability of planktonic cells in culture medium post-treatment corresponding to biofilms in Figure 3A and S8. Treatment p-values were calculated using a two-factor ANOVA test relative to the no treatment control. P-values are indicated with asterisks (1 asterisk = $p \leq 0.0332$, 2 asterisks = $p \leq 0.0021$, 3 asterisks = $p \leq 0.0002$, 4 asterisks = $p \leq 0.0001$). Data shown is an

average of 4 biological replicates and error bars represent standard deviation. [Abbreviations: Relative Fluorescence Units (RFU), CdTe-2.4 eV quantum dots (QD), Gentamicin (Gent)]

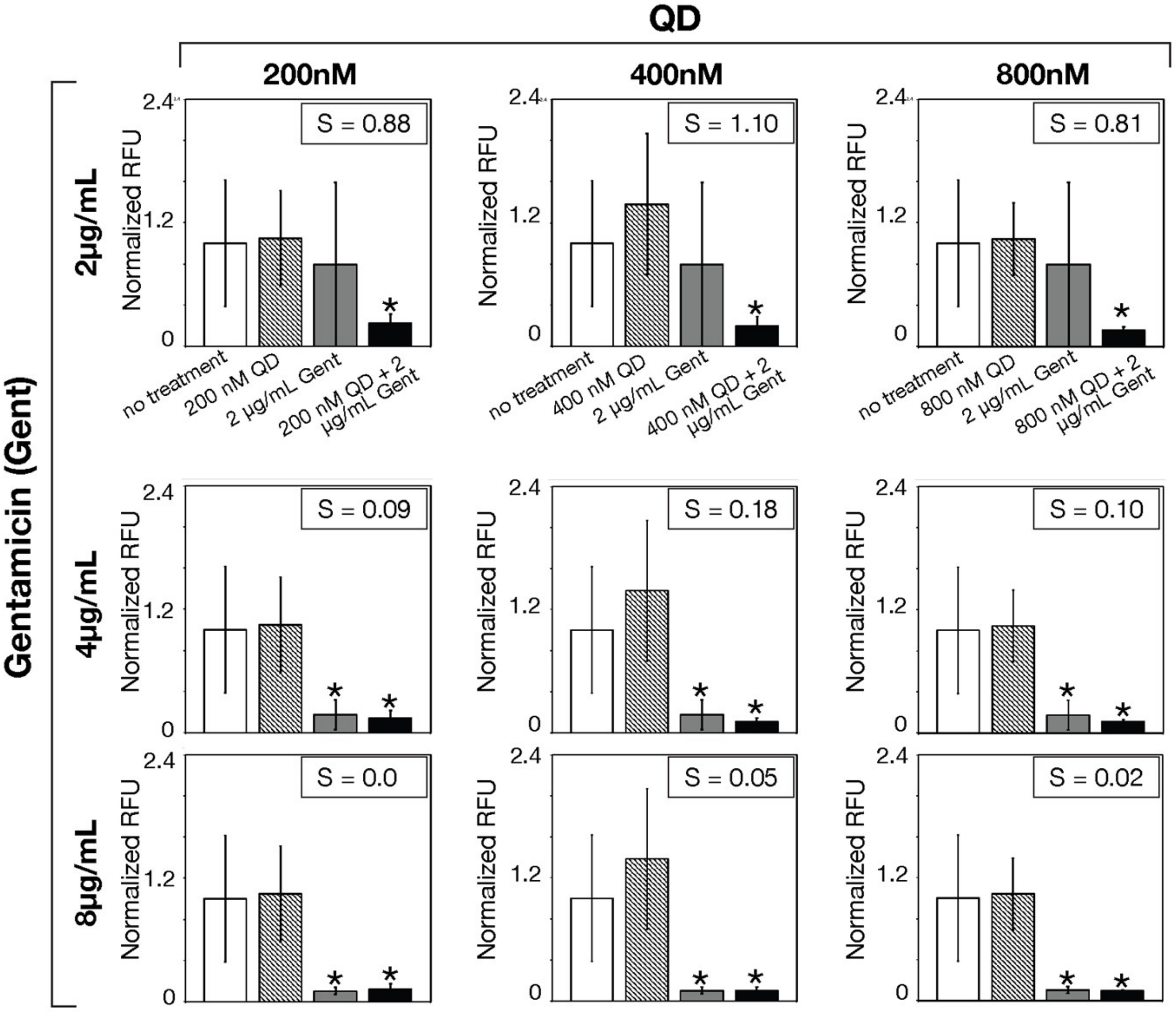

Supplemental Figure S10: PAO1 Combination-Monotherapy Comparison. This figure shows the full bar graphs of the relative fluorescence units (y-axis) of each combination therapy tested for PAO1 showing no treatment control (white), quantum dot (QD) (hatched), antibiotic (gray), and combination (black). Normalized resazurin fluorescence with respect to the no treatment control (1 asterisk: p-value ≤ 0.05). Synergy S-values (calculated by subtracting normalized relative fluorescence units of combination measurements from the product of its component monotherapies (S>0 indicates synergy, S<0 indicates antagonism)) are listed for each

combination respectively. None of the calculated S-values were statistically significant. Data is an average of five biological replicates and error bars represent the standard deviation.

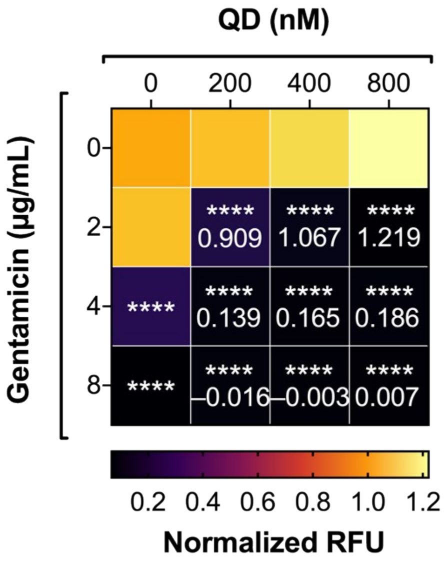

Supplemental Figure S11: PAO1 Planktonic Viability Combination Therapy Heatmap. Normalized RFU (ratio of fluorescence of treatment conditions relative to no treatment control) shows viability of planktonic cells in culture medium post-treatment corresponding to cells in Figure 3C. Synergy S-values calculated using a Bliss Independence model (subtracting RFU of combination therapy from the product of RFU of its component monotherapies) are listed for each combination in their respective cells. Treatment p-values were calculated using a two-factor ANOVA test relative to the no treatment control in the top left corner. P-values are indicated with asterisks (1 asterisk = $p \leq 0.0332$, 2 asterisks = $p \leq 0.0021$, 3 asterisks = $p \leq 0.0002$, 4 asterisks = $p \leq 0.0001$). Data shown is an average of 4 biological replicates and error bars represent standard deviation. [Abbreviations: Relative Fluorescence Units (RFU), CdTe-2.4 eV quantum dots (QD)]

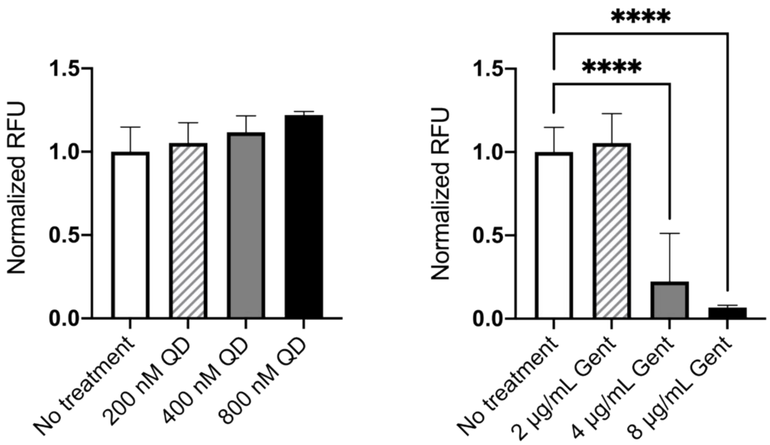

Supplemental Figure S12: PAO1 Monotherapeutic Planktonic Viability. Normalized RFU (ratio of fluorescence of treatment conditions relative to no treatment control) shows viability of planktonic cells in culture medium post-treatment corresponding to cells in Figure 3C. Treatment p-values were calculated using a two-tailed t test relative to the no treatment control. P-values are indicated with asterisks (1 asterisk = $p \leq 0.0332$, 2 asterisks = $p \leq 0.0021$, 3 asterisks = $p \leq 0.0002$, 4 asterisks = $p \leq 0.0001$). Data shown is an average of 4 biological replicates and error bars represent standard deviation. [Abbreviations: Relative Fluorescence Units (RFU), CdTe-2.4 eV quantum dots (QD), Gentamicin (Gent)]

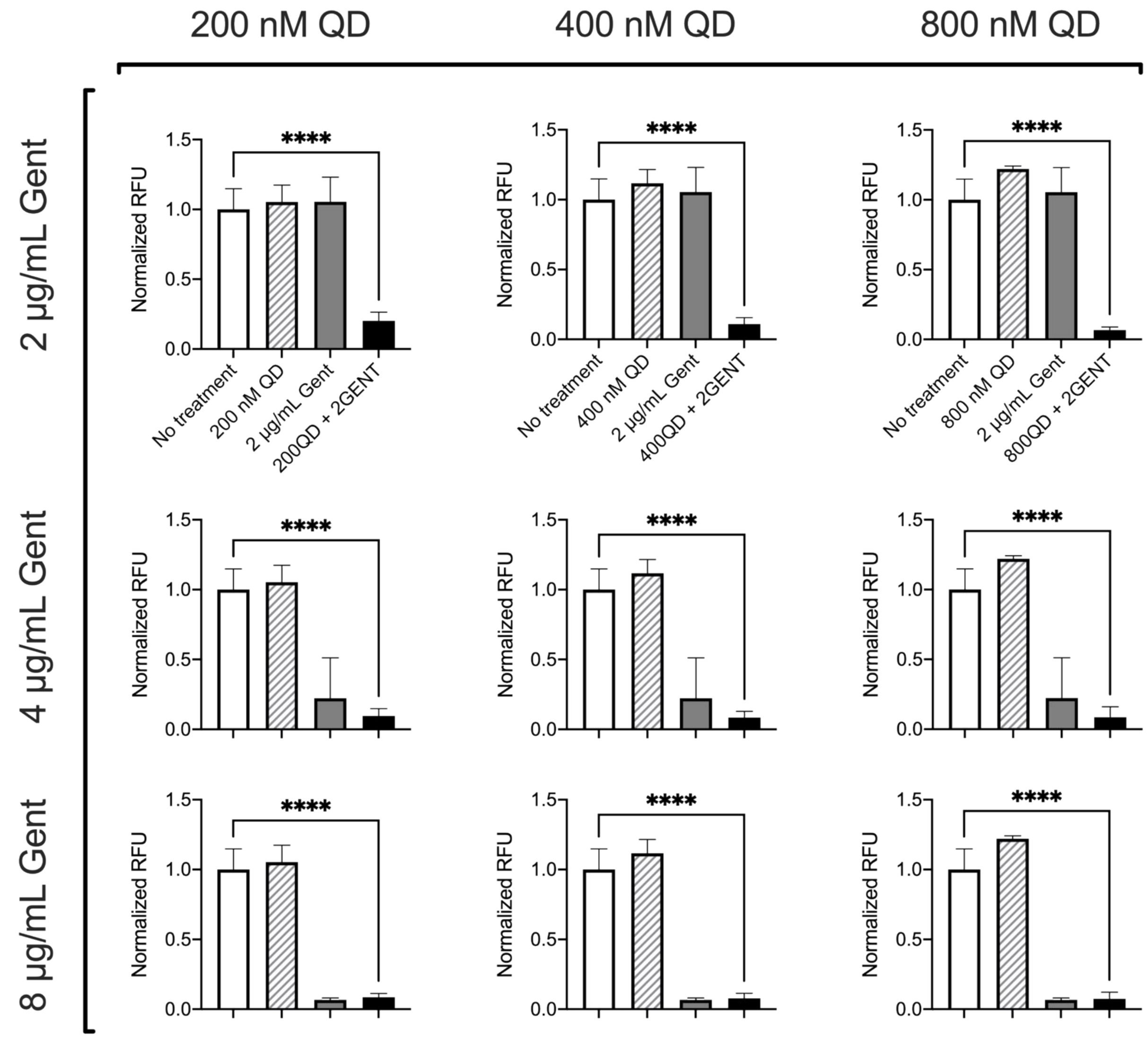

Supplemental Figure S13: PAO1 Planktonic Viability Post-Combination Therapy. Normalized RFU (ratio of fluorescence of treatment conditions relative to no treatment control) shows viability of planktonic cells in culture medium post-treatment corresponding to biofilms in Figure 3C and S12. Treatment p-values were calculated using a two-factor ANOVA test relative to the no treatment control. P-values are indicated with asterisks (1 asterisk = $p \leq 0.0332$, 2 asterisks = $p \leq 0.0021$, 3 asterisks = $p \leq 0.0002$, 4 asterisks = $p \leq 0.0001$). Data shown is an

average of 4 biological replicates and error bars represent standard deviation. [Abbreviations: Relative Fluorescence Units (RFU), CdTe-2.4 eV quantum dots (QD), Gentamicin (Gent)]

PA01					
			QD		
			200nM	400nM	800nM
Gentamicin	2μg/mL	*S*	0.88	1.1	0.81
		p	0.146	0.237	0.493
	4μg/mL	*S*	0.09	0.18	0.1
		p	0.129	0.176	0.171
	8μg/mL	*S*	0	0.05	0.02
		p	0.142	0.251	0.337

Supplemental Table S4: PAO1 Combination Treatment S-values and Corresponding p-values. Synergy S-values (calculated by subtracting normalized relative fluorescence units of combination measurements from the product of its component monotherapies ($S>0$ indicates synergy, $S<0$ indicates antagonism)) are listed for each combination respectively. Synergy p-values listed alongside the S-value were calculated with respect to the product of component monotherapies. Data is an average of five biological replicates and error bars represent the standard deviation.
